# A PKC-SHP1 signaling axis desensitizes Fcγ receptor signaling by reducing the tyrosine phosphorylation of CBL and regulates FcγR mediated phagocytosis

**DOI:** 10.1186/1471-2172-15-18

**Published:** 2014-05-07

**Authors:** Shweta Joshi, Alok Ranjan Singh, Muamera Zulcic, Donald L Durden

**Affiliations:** 1 UCSD Department of Pediatrics, Moores UCSD Cancer Center, University of California School of Medicine, San Diego, CA 92093, USA; 2 Division of Pediatric Hematology-Oncology, UCSD Rady Children’s Hospital, San Diego, CA, USA

**Keywords:** Fcγ receptors, PKC, CBL, CRKL, ITAM, Phagocytosis, SHP1

## Abstract

**Background:**

Fcγ receptors mediate important biological signals in myeloid cells including the ingestion of microorganisms through a process of phagocytosis. It is well-known that Fcγ receptor (FcγR) crosslinking induces the tyrosine phosphorylation of CBL which is associated with FcγR mediated phagocytosis, however how signaling molecules coordinate to desensitize these receptors is unclear. An investigation of the mechanisms involved in receptor desensitization will provide new insight into potential mechanisms by which signaling molecules may downregulate tyrosine phosphorylation dependent signaling events to terminate important signaling processes.

**Results:**

Using the U937IF cell line, we observed that FcγR1 crosslinking induces the tyrosine phosphorylation of CBL, which is maximal at 5 min. followed by a kinetic pattern of dephosphorylation. An investigation of the mechanisms involved in receptor desensitization revealed that pretreatment of U937IF or J774 cells with PMA followed by Fcγ receptor crosslinking results in the reduced tyrosine phosphorylation of CBL and the abrogation of downstream signals, such as CBL-CRKL binding, Rac-GTP activation and the phagocytic response. Pretreatment of J774 cells with GF109203X, a PKC inhibitor was observed to block dephosphorylation of CBL and rescued the phagocytic response. We demonstrate that the PKC induced desensitization of FcγR/ phagocytosis is associated with the inactivation of Rac-GTP, which is deactivated in a hematopoietic specific phosphatase SHP1 dependent manner following ITAM stimulation. The effect of PKC on FcγR signaling is augmented by the transfection of catalytically active SHP1 and not by the transfection of catalytic dead SHP1 (C124S).

**Conclusions:**

Our results suggest a functional model by which PKC interacts with SHP1 to affect the phosphorylation state of CBL, the activation state of Rac and the negative regulation of ITAM signaling i.e. Fcγ receptor mediated phagocytosis. These findings suggest a mechanism for Fcγ receptor desensitization by which a serine-threonine kinase e.g. PKC downregulates tyrosine phosphorylation dependent signaling events via the reduced tyrosine phosphorylation of the complex adapter protein, CBL.

## Background

Signal transduction events initiated by stimulation of Fc receptors are important to understanding processes such as immune reactions, inflammation, autoimmunity, and leukemic transformation. FcγRI, the high-affinity Fc receptor for monomeric IgG (CD64) and FcγRIIIA (CD16), the low affinity Fc receptor for IgG, are members of the immunoglobulin gene super family, which includes the T cell receptor, the B cell receptor, and Fc receptors such as the multi-subunit immune receptors (IRs) for IgE and IgG [1]. Importantly, both FcγRI and FcγRIIIA receptors signal through the 7 kd FcγRIγ subunit ITAM (termed the gamma subunit), whereas the FcγRIIA receptor contains a receptor intrinsic ITAM motif and the FcγRIIb receptor contains an immunoreceptor tyrosine inhibitory motif, ITIM [2]. Fc receptors are unique in that they do not possess intrinsic kinase activity, but mediate downstream signaling events through a conserved stretch of amino acids containing (YXXL), the immunoreceptor tyrosine based activation motif ITAM, which resides in the cytoplasmic region of the associated FcγRI gamma subunit or intrinsic to FcγRIIA receptor cytoplasmic domain [3].

The phosphorylated ITAM serves as a docking site for the recruitment of Syk/Zap-70 cytoplasmic nonreceptor protein tyrosine kinases (PTKs) as pre-requisite for their activation [4,5]. Fc receptor activation is associated with the rapid tyrosine phosphorylation of multiple cellular proteins including the adaptor protein, CBL, which is complexed with the gamma subunit of FcγRI and other FcγRs [6,7]. Tyrosine phosphorylation of CBL following Fcγ receptor stimulation has been implicated in intracellular signaling pathways via its interaction with several signaling molecules e.g. nonreceptor kinases, other adapter proteins [6,8,9]. The full-length c-CBL gene product, CBL is widely expressed in the cytoplasm and is known to consist of an amino terminal TKB domain, a ring finger domain and a carboxy terminal leucine zipper domain [10]. CBL possesses a number of proline rich motifs as well as several tyrosine residues in the C-terminus that bind kinases and adaptor proteins containing SH2 or SH3 domains (e.g., Nck, Grb2, Crk, CrkL, Syk, Fyn, and Lyn) that regulate guanine nucleotide exchange factors in mammalian cells [6,7,11,12]. The interaction of Syk with CBL (a multi-domain complex adaptor protein) [6,11,12] seems to be facilitated by some Src family kinases that can interact with proline rich PxxP motifs within the CBL protein by means of their SH3 domain [13]. The SH2- and SH3-domain containing CrkL adaptor protein is known to bind CBL at Y700 and Y774 a YxxP motif in human macrophages [14] as well as in normal T cells [15]. It has also been suggested that CBL can serve as a linker for phosphatidyl-inositol-3 kinase (PI-3Kinase) via Y731 site [16-18].

It has been shown recently that after TCR stimulation, CBL is phosphorylated not only on tyrosine, but also on serine, and that this post-translational modification regulates its interaction with 14-3-3 ζ-proteins [19]. The phorbol ester PMA induced the serine phosphorylation of CBL and induced its interaction with 14-3-3 ζ-proteins, implicating the protein kinase C family of serine/threonine kinases in CBL function. These reports prompted us to examine the signaling molecules that might be involved in mediating the attenuation of CBL tyrosine phosphorylation following Fcγ receptor activation. Protein kinase C (PKC) is a family of serine/threonine kinase that plays critical role in the regulation of differentiation and proliferation of many cell types in the presence of diverse stimuli [20,21]. The PKC family consists of at least 11 isoforms that can be classified into three main subfamilies based on their homology and cofactor requirements for activation: conventional PKCα, βI, βII and γ are diacylglycerol (DAG) and calcium-dependent; novel PKCδ, ε, θ and η are DAG-dependent and calcium-independent; and finally a typical PKCζ, λ and ι are dependent only on phospholipids [22]. Studies indicate that PKC is also important during myeloid and lymphoid activation. Recent evidence suggests that Protein Kinase C (PKC) may desensitize tyrosine kinase linked receptors by altering the tyrosine phosphorylation state of the receptor-signaling complex [23]. However the mechanism is not yet clearly defined.

Previous work done by our laboratory [24] and other groups [25] have reported that tyrosine phosphorylation plays a key role in Fcγ receptor mediated phagocytosis that is essential for activation of PI-3 kinase. Phagocytosis of pathogens by macrophages initiates the immune response, which in turn orchestrates the adaptive immune response. Phagocytosis is associated with a variety of cellular responses, including a rise in [Ca^++^] [26], activation of PKC [27], generation of respiratory burst, release of arachidonic acid [28] and tyrosine phosphorylation [29-31]. During FcγR mediated phagocytosis (specific opsonization with antibodies), FcγIIIA and IIB receptors are co-cross linked by Fc portion of IgG coated surface of the foreign invaders. Activation of macrophages through Fc receptors leads to activation of non-receptor protein tyrosine kinase, Src and Syk families [32-35]. Recently Crowley and Kiefer [36,37] have reported that macrophages derived from Syk deficient mice display defect in phagocytosis of IgG coated particles indicating an important role of Syk kinase in phagocytosis. In our earlier reports, we reported the involvement of Src family kinase in the tyrosine phosphorylation of CBL and Syk which are required for the formation of ITAM/Syk/CBL complex to initiate phagocytic response. A recent report suggested that negative regulation of class IA PI-3 kinase by PKC δ limits Fcγ receptor mediated phagocytosis in macrophages [38]. It has also been reported by our laboratory [24] and other groups [39-41] that SHP1 plays a key role in Fcγ receptor mediated phagocytic signal transduction. In the present study, we have investigated the interplay between PKC and SHP1 to regulate the deactivation of the Fcγ receptor, which leads to inhibition of phagocytic signal transduction cascade. Herein, we demonstrate that PKC activated by PMA desensitizes FcγR signaling an effect which is correlated with the reduced receptor-induced tyrosine phosphorylation of CBL. The effect of PKC on FcγR mediated phagocytosis, and downstream alteration in signaling are both dependent on the transfection of catalytically active protein tyrosine phosphatase, SHP1. These results provide new insight into potential mechanisms by which PKC may downregulate tyrosine phosphorylation dependent signaling events to regulate receptor desensitization.

## Results

### Kinetics of CBL tyrosine phosphorylation and dephosphorylation upon FcγR1 stimulation

**Figure 1:**
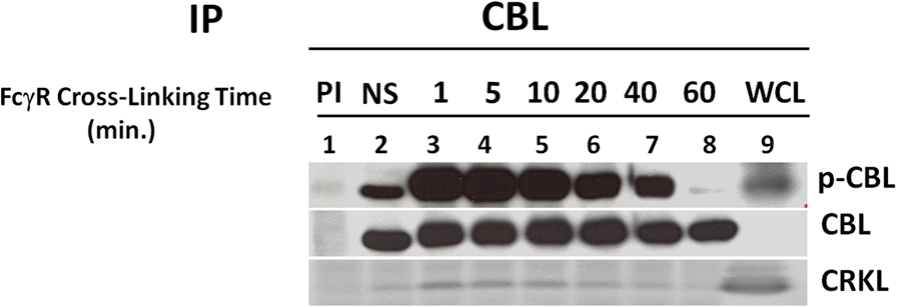
**Time course analysis of tyrosine phosphorylation and dephosphorylation of CBL and its association with the CRKL adapter protein following FcγR1 activation.** Anti-phosphotyrosine immunoblot was performed on CBL immunoprecipitated from U937 cells with anti-phosphotyrosine antibody (4G10). Lane 1, preimmune (1 mg/ml rabbit IgG) and lane 2, resting U937 cells (NS). FcγR1 stimulation [32.2mAb F(ab)_2_] and crosslinking with rabbit anti mouse antisera to U937 cells were done for 1, 5, 10, 20, 40 & 60 min., lane 3 to 8 respectively. Lane 9, whole cell lysate (WCL). This blot was again reprobed with anti-CBL and anti-CRKL antibody to confirm equal precipitation of CBL in all lanes and its association with CRKL. This experiment was repeated three times.

In order to study the effect of Fcγ crosslinking on tyrosine phosphorylation of CBL, U937IF cells were used which express two ITAM containing activating receptors FcγRI and FcγRIIA. We focused our studies on FcγRI because monocytes express this receptor under inflammatory conditions and we have used this system to examine FcγRI signaling downstream of the ITAM [42,43]. Our results show the kinetics of phosphorylation and dephosphorylation of CBL immunoprecipitated from resting and FcγR1 stimulated U937IF cells differentiated with human interferon gamma (250 U/ml) for 5 days. FcγR1 was stimulated with anti-FcγR1 [32.2 F (ab)_2_] at 37°C for 30 min. and cross linked with secondary antibody (rabbit F(ab)_2_ fragment to mouse IgG) for different time periods at 37°C. CBL (120 KD) displays a basal level of tyrosine phosphorylation in resting cells (Figure 1, lane 2) and reached peak within 1 to 5 min. of receptor aggregation (Figure 1, lane 3 and 4). Twenty min. after FcγR1 stimulation, the dephosphorylation of CBL begins (Figure 1, lane 6) and by 60 min. CBL is completely dephosphorylated (Figure 1, lane 8).

### CBL-CRKL association is correlated with the tyrosine phosphorylation of CBL upon FcγR1 stimulation

The results shown in Figure 1 demonstrate that CBL undergoes tyrosine phosphorylation followed by dephosphorylation upon FcγR1 aggregation in U937IF cells. Previous reports from our laboratory showed that upon FcγR1 stimulation, CBL is tyrosine phosphorylated and concomitantly bound with CRKL [12,44]. The membrane was cut and reprobed with anti-CBL and anti-CRKL antibody to show that small amount of CRKL was associated with CBL in resting state. An increase in CBL-CRKL binding was noted within the first 1 min. (Figure 1, lane 3) to 5–10 min. (Figure 1, lane 4 and 5) of stimulation. By 20–30 min. after FcγR1 stimulation CBL-CRKL interaction began to decrease and minimal binding was observed by 60 min. (Figure 1, lane 8). From these data, we can conclude that CBL-CRKL interaction in myeloid cells correlates with the tyrosine phosphorylation of CBL upon FcγR1 stimulation.

### PMA stimulation reduced the tyrosine phosphorylation of CBL

**Figure 2:**
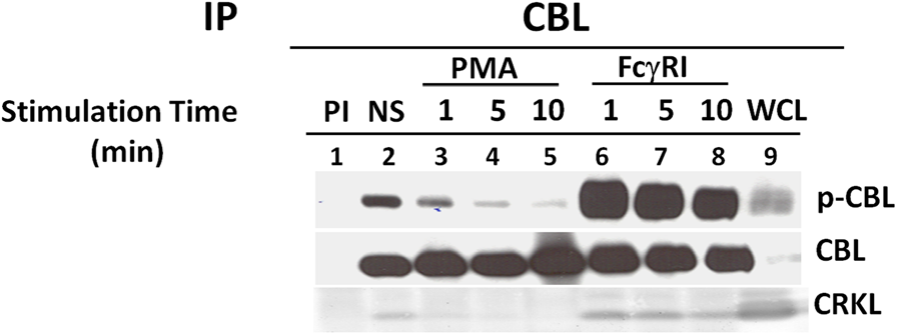
**PMA treatment reduced the tyrosine phosphorylation of CBL and dissociation of binding of CRKL to CBL.** Immunoprecipitations of U937 cell lysates were performed with anti-CBL antibody after PMA or FcγRI stimulation. Lane 1 & 2 indicates preimmune and resting state (NS) respectively. Lane 3, 4 & 5, treated with PMA (200 ng/ml) for 1, 5 and 10 min. respectively. Lane 6, 7 & 8, stimulated for FcγR1 with 32.2 F(ab)_2_ and cross linked with secondary antibody (rabbit anti mouse F(ab’)2) for 1 , 5 and 10 min. respectively. Whole cell lysate was added in lane 9 as positive control (WCL). Immunoprecipitated proteins were resolved in 10% SDS-PAGE, blot on nitrocellulose membrane and probed with anti-phosphotyrosine antibody (4G10). This blot was again reprobed with anti-CBL and anti-CRKL antibody. This experiment was repeated three times.

It has been reported by Fernandez et.al. [23] that treatment using PMA decreases tyrosine phosphorylation of CBL in T-cells. In this present study, we have found that PMA stimulation significantly decrease CBL phosphorylation in myeloid cells (Figure 2). As shown in Figure 2, CBL immunoprecipitated from resting state U937IF cells exhibited relatively low level of tyrosine phosphorylation, which was significantly increased upon FcγR1 stimulation. When cells were stimulated with PMA (200 ng/ml), the tyrosine phosphorylation of CBL is markedly decreased after 5 min. (Figure 2, lane 4) and there is no CBL phosphorylation after 10 min. (Figure 2, lane 5). These results establish that the CBL adapter protein underwent a significant decrease in tyrosine phosphorylation after PMA treatment.

A prominent feature of FcγR1stimulation in myeloid cells is the transient association of CRKL with CBL [12]. Figure 2 shows that the association of CRKL with CBL was increased by FcγR1 stimulation (Figure 2, lane 6, 7 and 8) and this interaction is reduced by PMA treatment (Figure 2, lane 3, 4 and 5).

### PKC activation attenuates tyrosine phosphorylation of CBL

**Figure 3:**
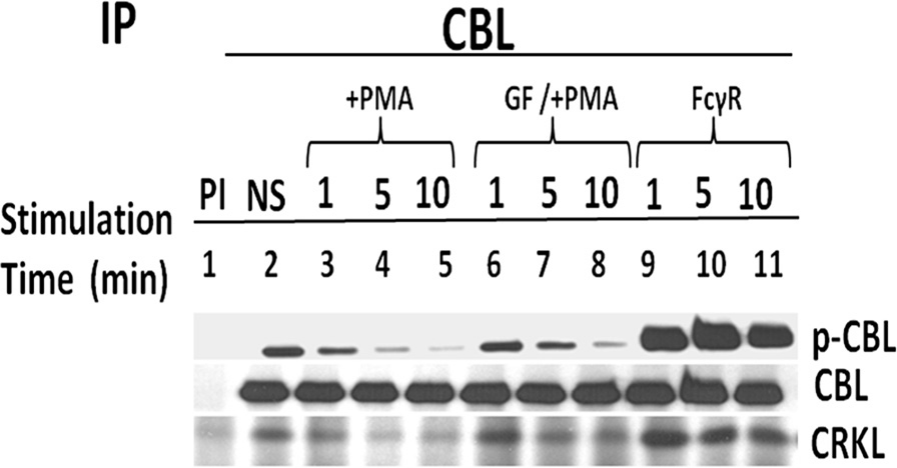
**Specific PKC inhibitor (GF109203X) maintains the basal tyrosine phosphorylation state of CBL.** Human IFN γ differentiated U937 cells were treated with or without GF 109203X (2.5 μM) on ice for 15 min. and then stimulated with PMA (200 ng/ml) for 1, 5 & 10 min. at 37°C. Cell lysates were prepared and immunoprecipitated (IP) with anti-CBL antibody. Lane 1 represents precipitation with preimmune antisera. Lane 2, no stimulation. Lane 3, 4 & 5 and 9, 10 & 11 represent U937 cells stimulated with PMA (200 ng/ml) or 32.2 F(ab)2 for 1, 5 & 10 min. respectively. Lane 6, 7 & 8, represents cells preincubated with GF 109203X followed by PMA stimulation. This blot was reprobed with anti-CBL and anti-CRKL antibody.

To determine the specific effect of PKC on tyrosine phosphorylation of CBL, U937IF cells were pre-incubated with a specific PKC inhibitor, GF109203X, followed by PMA treatment. Our data suggest that CBL phosphorylation was maintained for 1 to 5 min. (Figure 3, lane 6 and 7) in GF109203X treated cells pre-incubated with PMA in comparison to cells treated with PMA alone (Figure 3, lanes 3, 4 and 5). In addition to CBL phosphorylation, CBL-CRKL association was also maintained in cells pre-incubated with GF109203X.

**Figure 4:**
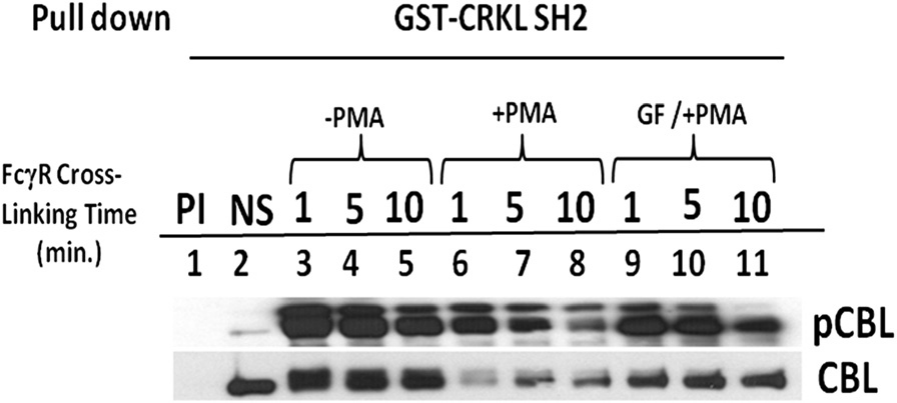
**GST-CRKL-SH2 pull down experiment showing the effect of PMA on CBL-CRKL dissociation and CBL dephosphorylation.** U937IF cell lysates were prepared and were precipitated with GST fusion protein as described in Methods. 10 μg of GST fusion protein was used for in*vitro* pull down experiments. 50 μl of glutathione-sepharose beads (prewashed with extraction buffer) was added to each sample. GST-Crkl-SH2 fusion (lanes 2–11) protein was used to precipitate associated protein with or without PMA treatment in FcγRI stimulated cell lysate. Lane 1, precipitated with GST alone. Lane 2, no stimulation, Lane 3, 4 & 5, stimulated cell lysate (stimulated with 32.2 F(ab)_2_ antibody and cross linked with secondary antibody for 1, 5 and 10 min. Lane 6, 7 and 8, cells were treated with PMA (200 ng/ml) for 5 min. followed by FcγR1 stimulations, lane 9, 10 and 11, cells were preincubated with GF109203X (2.5 μM) for 15 min. on ice and then treated with PMA (200 ng/ml) for 5 min. followed by FcγR1 stimulations. Proteins bound to GST fusion protein were resolved in 10% SDS-PAGE, blotted on nitrocellulose membrane and probed with phosphotyrosine antibody (4G10). This blot was reprobed with anti-CBL antibody. This experiment was repeated two times.

In order to study CBL-CRKL interaction in U937IF cells following FcγR1 stimulation, we performed pull-down experiments with the GST-CRKL-SH2 domain under conditions of FcγR1 stimulation alone and pretreatment with PMA. Similar to data shown in Figure 2, we observed a basal level of tyrosine phosphorylation of CBL in GST-CRKL-SH2 pull down from resting state (Figure 4, lane 2). Within 1 min. of FcγR1 stimulation a marked increase was seen in amount of tyrosine phosphorylated protein bound to CRKL (Figure 4, compare lane 2 with 3). Pretreatment with PMA followed by FcγR1 cross linking showed decreased amount of phosphorylated CBL pulled down with CRKL-SH2 (Figure 4, lane 6, 7 and 8). To provide further evidence for the role of PKC in tyrosine phosphorylation of CBL through Fcγ receptor desensitization, we performed GST-CRKL-SH2 pull-down experiment with cells pretreated with GF109203X, then stimulated with PMA for 5 min followed by cross linking of FcγR1 at different time periods. Pretreatment with GF109203X partially rescued the PMA induced tyrosine dephosphorylation of CBL (Figure 4 compare lane 9, 10 and 11 with lane 6, 7 and 8). The protective effect of GF109203X on CBL dephosphorylation appeared concomitant to CBL-CRKL association. In addition, we observe a 130 kd tyrosine phosphorylated protein which binds to the CRKL-SH2 domain upon FcγR1 crosslinking and appears to undergo a reduced level of tyrosine phosphorylation upon PMA treatment in U937IF cells (Figure 4). The effect of the PKC inhibitor on tyrosine phosphorylation state of CBL as well as CBL-CRKL interaction suggest that PMA acts through an effect upon tyrosine phosphorylation of CBL following FcγR stimulation.

### PMA reduced the tyrosine phosphorylation state of CBL and abrogates phagocytosis in J774 A.1 cells stimulated with IgG sensitized sheep RBC

**Figure 5:**
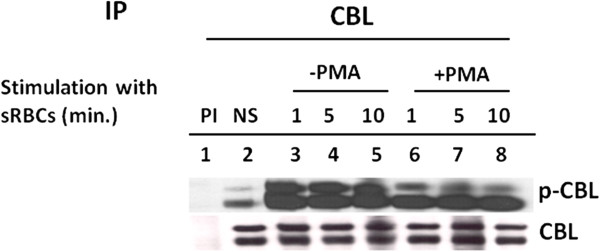
**PMA induces dephosphorylation of CBL in mouse macrophage cell line (J774A.1) stimulated with sensitized sheep RBC.** CBL immunoprecipitation was done in J774A.1 cells stimulated with sensitized (IgG coated) sheep RBCs at 37°C in a time dependent manner. Anti-phosphotyrosine blot showing CBL immunoprecipitation. Lane 1, preimmune and lane 2, resting condition (NS). Lane 3, 4 & 5, cells were stimulated with sensitized sheep RBCs for 1, 5 and 10 min. Lane 6, 7 & 8, preincubated with PMA (200 ng/ml) for 5 min. at 37°C followed by FcγR stimulation for 1, 5 and 10 min. respectively.

**Figure 6:**
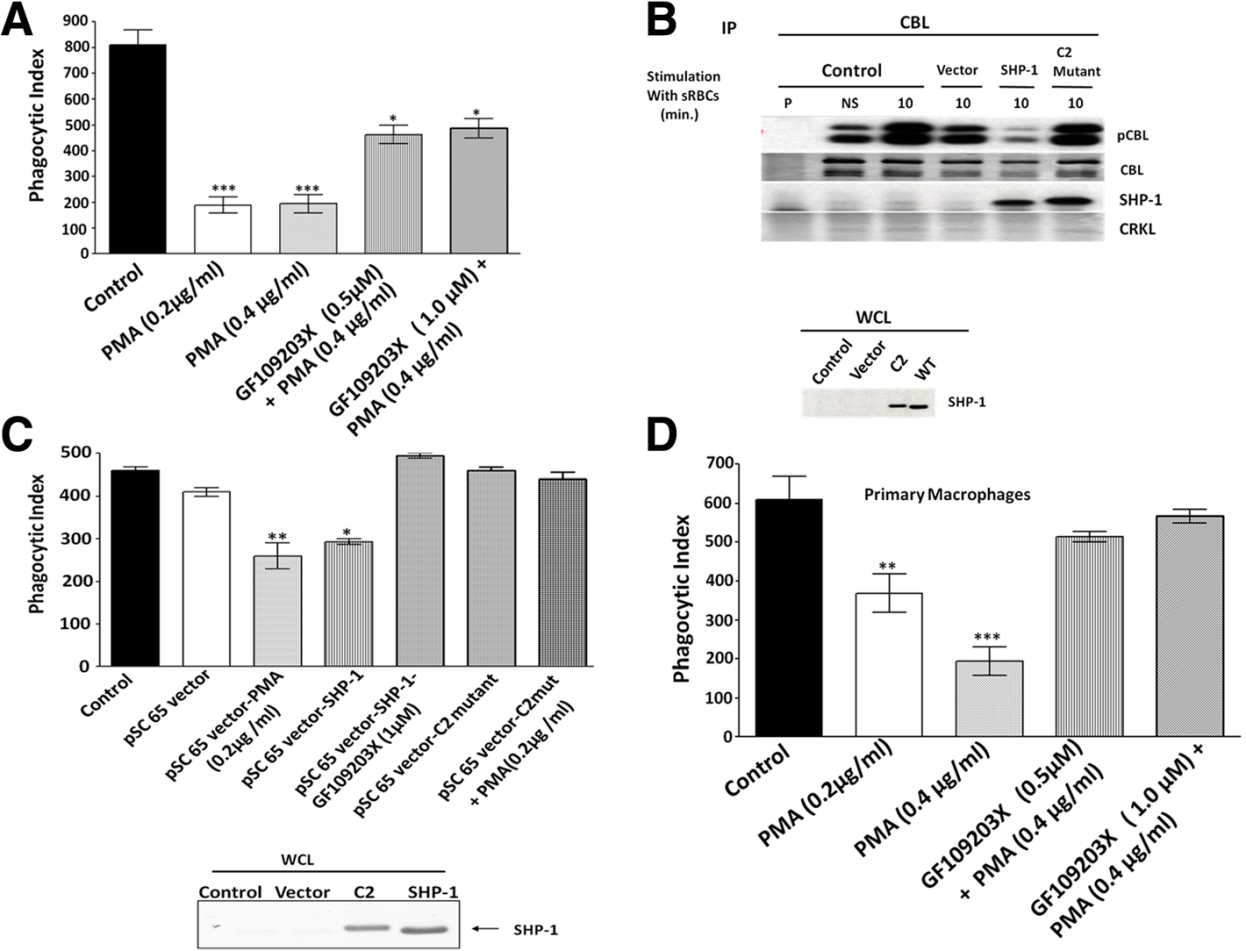
**Fcγ Receptor induced phagocytic activity is decreased following the treatment by PMA in J774A.1 cell line. (A)** For PMA treatment, cells were pre incubated with PMA (0.2 μg/ml or 0.4 μg/ml) for 15 min. before the onset of phagocytosis. In other set, cells were pre incubated with GF109203X for 15 min. followed by PMA (0.4 μg/ml) treatment. To initiate phagocytosis, media was replaced by 1ml IgG coated sheep RBCs and incubated for 30 min. at 37^o^C. **(B)** Effect of over expression of SHP-1 on CBL dephosphorylation.. J774A.1 cells were infected either with empty vector recombinant virus (pSC 65) or wild type SHP-1 (MOI 2 pfu/cell for overnight) or with C2 mutant of SHP-1. P and NS represents pre-immune and non-stimulation respectively, non infected serve as control. Except P and NS conditions, all cells were stimulated with sensitized sheep RBCs for 10 min. Immunoblots were reprobed with anti-CBL, anti-SHP-1 and anti-CRKL antibody. Lower panel shows the Western blot analysis of over expression of SHP-1 and C2 mutant in J774A.1 cells. **(C)** J774A-1 cells were infected with pSC 65or recombinant vaccinia for the expression of C2 mutant or SHP-1 at an MOI of 2 pfu / cell for 4 hours at 37^o^C as described in Methods. Western blot analysis confirms equivalent level of over expression of SHP-1 and C2 mutant in J774-1 cells. **(D)**. Peritoneal macrophages isolated from C57BL/6 mice were pre incubated with PMA for 15 min. before the onset of phagocytosis. In other set, cells were pre incubated with GF109203X (above mentioned conc.) for 15 min. followed by PMA (0.4 μg/ml) treatment and phagocytosis was performed as described above. Each bar represents mean ± SD, n=3. *P <0.05, **P <0.01 and ***P <0.001 vs. control (t-test) All these experiments were performed three times.

Since several studies have recently reported the requirement for specific tyrosine kinases, Syk and Src family kinases in phagocytosis mediated events [32,33,36,45], we presumed that dephosphorylation of specific cell protein on specific tyrosine phosphorylation sites may negatively regulate phagocytosis. We previously reported that Fcγ receptor cross-linking induces tyrosine phosphorylation of the complex adapter protein CBL [11]. More recent data from our laboratory provide evidence that CBL is required in FcγR mediated phagocytic response [24]. These observations prompted us to study the role of tyrosine phosphorylation of CBL in the induction of phagocytic signaling. To address this question, we have studied the tyrosine phosphorylation of CBL in J774 A.1 cells stimulated with IgG sensitized sheep RBC. Figure 5 shows that tyrosine phosphorylation of CBL is attenuated in PMA pre-treated cells upon Fcγ receptor stimulation (lane 6, 7 and 8). Phagocytosis of IgG coated sRBC (Figure 6A) was found significantly inhibited following treatment of PMA in J774A.1 cells. This inhibitory effect of PMA was blocked by the PKC inhibitor, GF109203X (Figure 6A). These data suggest that FcγR induced phagocytic event is regulated by PKC. Previous work in our laboratory demonstrated that SHP-1 overexpression inhibits FcγR mediated phagocytosis compared to no effect of a catalytically dead mutant of SHP-1 and heterologous overexpression of wild type SHP-1 in J774 A.1 enhances the dephosphorylation of CBL [24] . These results combined with our observation that PMA induced the dephosphorylation of CBL prompted us to examine the crosstalk between PKC and SHP-1 on FcγR mediated downstream event. To execute these experiments, J774A.1 cells were infected either with empty vector recombinant vaccinia virus (pSC65) or recombinant vaccinia virus for the expression of wild type SHP-1 or catalytically dead SHP-1 at an MOI 2 pfu/cell for 4 hours at 37°C. The result shows that effect of SHP-1 overexpression on phagocytosis is blocked by the inhibition of PKC (treatment with GF109203X) (Figure 6C). Importantly, PMA has no effect on Fcγ receptor mediated phagocytosis when cells were overexpressing the catalytically dead SHP1 (C124S). Hence, we suggest that PKC desensitizes Fcγ receptor in a SHP1 tyrosine phosphatase dependent manner resulting in the dephosphorylation of CBL an effect which blocks the downstream phagocytosis response.

To further validate the role of PKC in FcγR dependent phagocytosis, we performed experiments in primary murine macrophages (Figure 6D). Peritoneal macrophages isolated from mice were treated as shown in Figure 6A, pulsed with PMA or PMA + PKC inhibitor followed by incubation with anti-SRBC IgG opsonized SRBCs. Similar to results observed in J774 cells, PMA was noted to inhibit phagocytosis 70%, an effect that was reversed by the treatment with the PKC inhibitor, GF109203X (Figure 6D). These results closely replicate our findings in the J774 murine macrophage cell line (Figure 6A).

### PKC blocks FcγR1 mediated Rac activity

**Figure 7:**
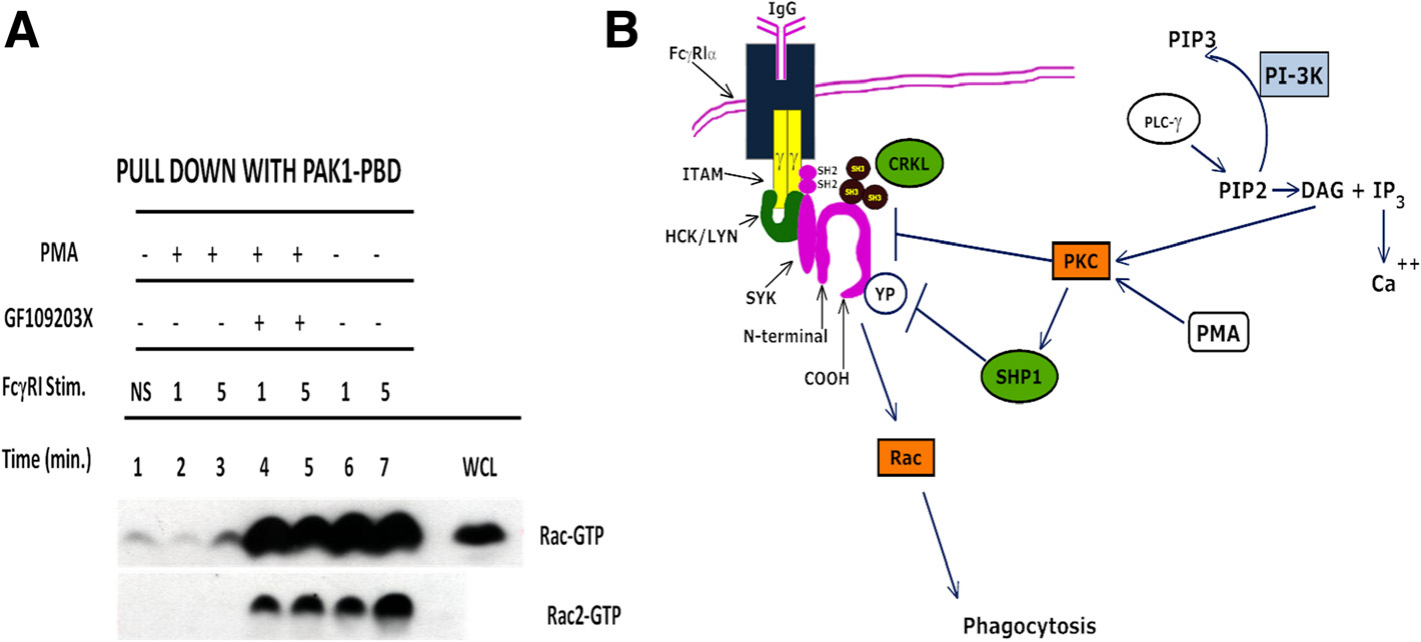
**FcγR1 activates Rac. (A)** The activation of total Rac and Rac2 was measured following FcγR1 stimulation in U937 cells (See Methods) using GST-PAK1_CRIB domain pull down assay. U937 cells were treated with PMA or PKC inhibitor (GF109203X) to determine the effect of PKC on Fcγ receptor desensitization. The protein bound to GST-PAK1-CRIB is resolved on SDS-PAGE and immunoblotted with antibody for total Rac. Same experiment was repeated and immunoblotted with antibody specific for Rac2. Lane 1, no stimulation; lane 2 & 3, preincubated with PMA (200 ng/ml) for 5 min. at 37**°C** followed by FcγR stimulation for 1 and 5 min. respectively, lane 4 & 5, cells were preincubated with GF 109203X (2.5 μM) for 15 min. on ice and then treated with PMA (200 ng/ml) for 1 & 5 min. followed by FcγR1 stimulations, lane 6 and 7, FcγR1 stimulation (alone) on Rac-GTP levels. The experiment was repeated three times. **(B)** Graphic representation of the signaling mechanism reported in the present study.

First, we performed experiments to confirm that FcγR stimulation induces the activation of the small GTPase, Rac. We then treated U937IF cells with PKC inhibitor, GF109203X or activator, PMA to examine the effect of PKC on Rac activation. From these data (Figure 7), we conclude that PKC induces dephosphorylation of CBL potentially through SHP1 and this is associated with a block in the downstream activation of Rac-GTPase activity. This observation is consistent with our published work showing in an heterologous FcγR/SYK reconstituted COS cell system that SHP1 and not SHP2 can regulate FcγR activation of Rac and this is correlated with marked inhibition of FcγR dependent phagocytosis [24]. Interestingly, inhibition of Rac activation following PMA treatment was directly correlated with the inhibition of CBL phosphorylation which corresponded with a marked inhibition of FcγR mediated phagocytosis in the J774 system and decrease association of CBL with CRKL. Figure 7B shows a schematic representation of the signaling pathway elucidated in this report.

## Discussion

Fc receptor activation is associated with rapid tyrosine phosphorylation of CBL adaptor protein, which is complexed via Syk kinase and the γ-subunit of FcγRI [6,7,14]. But the interplay between the key signaling molecules which dephosphorylate CBL upon receptor desensitization and hence the deactivation of the phagocytic signalsome are mostly unknown. In the present study, we identified an interaction between PKC and SHP1 which appears to regulate the deactivation of Fcγ receptor leading to the inhibition of the phagocytic signal transduction cascade.

Tyrosine phosphorylation is a critical event for the regulation of signal transduction, cell growth, differentiation and development. Tyrosine phosphorylation of CBL following Fcγ receptor stimulation has been implicated in intracellular signaling pathways via its interaction with several signaling molecules. Tyrosine phosphorylation of CBL has also been demonstrated upon stimulation through T cell receptor [15,46], B cell antigen receptor [47,48] granulocyte-macrophage colony stimulating factor and erythropoietin receptor [49], suggesting that CBL plays an important role in multiple antigen receptor and mitogen receptor associated tyrosine kinase activation pathways in hematopoietic cells.

The increased tyrosine phosphorylation of cellular proteins that occurs after FcγR stimulation is a transient process, which is regulated by the combined action of protein tyrosine kinases and phosphatases. Importantly, once a protein is phosphorylated *in vivo* on a tyrosine residue, this **covalent** phosphotyrosine moiety within a given intact protein can only be reversed by the action of a protein tyrosine phosphatase i.e. dephosphorylation (Figure 1). Interestingly, the peak of protein tyrosine phosphorylation in response to Fc receptor activation was observed only after 1 to 5 min. and decreased in next 10–20 min., which suggests that a tyrosine phosphatase was activated following Fcγ receptor engagement. Relatively less work has been done regarding the receptor deactivation mechanisms, including the regulation of phosphatase activity. Our results suggest that tyrosine dephosphorylation of proto-oncoprotein CBL is associated with receptor desensitization and leads to abrogation of the downstream phagocytic signal. These results raise the possibility that a specific tyrosine phosphatase is involved in deactivating the ITAM signaling cascade. Our previous findings [24] and the results observed in the present study indicate that CBL is a substrate for SHP1 and PMA can potentially induce the activation of SHP1. This dephosphorylation of CBL abrogates CBL-CRKL interaction following Fcγ receptor engagement. It has been also suggested that CBL-CRKL interaction is mediated through YXXP motif at the C terminal end of CBL (tyrosine 774) [12]. Our mass spectrometry data also confirmed that PMA abrogates CBL-CRKL interaction due to dephosphorylation of CBL at Tyr^774^ (unpublished observation). Based on these data, we suggest that PMA (probably through SHP1) targets the tyrosine dephosphorylation of CBL at Tyr^774^ which leads to loss of CBL-CRKL interaction. Several groups have demonstrated that CRKL interacts via its N-terminal SH3 domain with guanine nucleotide exchange factors C3G and SOS [50-52] suggesting that CRKL activates small G-protein signaling pathways. In Jurkat T cells, CBL through its interaction with CRKL protein becomes coupled to a guanine nucleotide exchange factor. These findings strongly suggest the possibility that tyrosine phosphorylation of CBL may provide one mechanism to link upstream tyrosine kinase to small G protein regulation. These finding are consistent with the result that PMA abrogates ITAM induced Rac-GTP activity (Figure 7), a biochemical phenomenon that has been implicated in the regulation of cytoskeleton rearrangement and phagocytosis.

PMA is known to activate PKCs by binding to their cysteine rich domain and facilitating their translocation to plasma membrane [53-55]. Using PKC specific inhibitor GF109203X (inhibits both classical and novel PKCs) the inhibitory effect of PMA on phosphorylation of CBL and phagocytosis was reversed, indicating the involvement of PKC in this phenomenon. The inhibition of PKC activity by GF109203X, leads to higher tyrosine phosphorylation of other cellular proteins. It has also been reported by others that PMA induces dephosphorylation of Shc in T cells and this can be reversed by GF109203X [23] indicating that PKC activation modulates the tyrosine phosphorylation state of other cellular protein besides CBL. It has also been reported previously by others that PMA causes serine/threonine phosphorylation of growth factor receptor and serine phosphorylation of IRS1 resulting an inhibition of receptor protein tyrosine kinase activity [56,57]. Liu et al. [19] have reported that PMA induces the serine phosphorylation of CBL and promote the association of Tau isoform of 14-3-3 and this serine phosphorylation of CBL suppressed its tyrosine phosphorylation by tyrosine kinase inhibition, however the mechanism is not yet known. It has also been reported that PKC does not bind to CBL directly [58], raising the possibility that PKC might indirectly regulate tyrosine phosphorylation of CBL by bringing a tyrosine phosphatase into the signalsome, by activating a specific PTP or by altering substrate availability to kinases and/or phosphatases. Our results provide evidence that PKC controls the tyrosine phosphorylation state of CBL via a mechanism that somehow requires the protein tyrosine phosphatase activity of SHP1.

In the present study, we examined the role of PKC in the control of Fcγ receptor mediated phagocytosis. The data presented here demonstrate that Fcγ receptor mediated phagocytosis was markedly inhibited by exposure of cells to PMA, which was associated with CBL tyrosine dephosphorylation and downstream inhibition of Rac-GTP activation. The literature contains conflicting reports concerning the role of PKC in phagocytosis. Pharmacological inhibition or expression of dominant negative isoforms of PKC reduced phagocytosis in several systems [59-61]. However, the precise role of the particular PKC isoforms involved in phagocytosis remains unclear. Involvement of PKC activity in complement receptor-mediated phagocytosis has been clearly demonstrated [62]. In the case of FcγR-mediated phagocytosis, data are more complex. Differences in these reports regarding the involvement of PKC may be a result of the differential role of various PKC isoforms in phagocytosis [63]. In U937 monocytes, it was found that FcγRI engagement leads to an increase in PKC activity that is Ca^2+^-independent and corresponds to translocation to the membrane of the PKC isoforms δ, ϵ, and ζ [64]. In U937-differentiated macrophages, FcγRI engagement leads to PKC activity that is Ca^2+^-dependent and corresponds to membrane translocation of the conventional PKC isoforms α, β, and γ [64,65].

Zheleznyak and Brown [66] have found that activation of PKC is an early signal required for Fc receptor mediated phagocytosis in human monocytes. It is possible that PKC may play a different role in Fcγ receptor signaling and phagocytosis at different time points following receptor engagement as is true for many signaling molecules. We [24] and others have demonstrated that tyrosine phosphorylation is a critical signaling event that underlies Fcγ receptor mediated phagocytosis in mouse macrophages and the formation of tyrosine phosphorylation coincides with the appearance of F-actin beneath the phagocytic cup. [29,62,67]. They reported that genestein (tyrosine kinase inhibitor) but not inhibitors of protein kinase C, block the ingestion process during phagocytosis. In agreement with these data, we observed that PMA induces Fc receptor mediated rapid CBL dephosphorylation which coincides with abrogation of phagocytosis. It has also been reported by Romanova et al. [68] that Rac1 is necessary for the IL3 induced assembly of membrane ruffles in Baf3 (human pre-B lymphoid) cell line and PMA dissolves the actin formed membrane ruffles and round the cells in presence of IL-3. Rac is directly involved in actin polymerization/formation of lamellipodia. It plays an important role in engulfment in phagocytosis. Our results suggest that PMA induced a dephosphorylation of CBL which is associated with a block in the downstream signaling cascade, the conversion of Rac-GDP to Rac-GTP, an event that is essential for the phagocytic response.

Tyrosine phosphorylation is controlled by the coordinated action of PTKs (protein tyrosine kinase) and protein tyrosine phosphates (PTPs) [69-71]. The work from our laboratory has reported [24] that SHP1 inhibits Fcγ receptors mediated phagocytosis by altering the phosphorylation state of CBL and blocked Rac-GTP. In macrophages, SHP1 also selectively regulates the tyrosine phosphorylation of Stat1 and Jak1 while leaving Tyk2 and Stat2 unaffected [72].

Herein, we present evidence that PKC negatively regulates the tyrosine phosphorylation state of the CBL adapter protein and inhibits FcγR dependent ITAM signaling and phagocytosis. Our experiments performed with heterologous expression of SHP1 vs. the phosphatase-dead SHP1 support the notion that this PKC mediated event is dependent upon SHP1 phosphatase activity. Previous reports from our laboratory and others have confirmed a role of CBL in the regulation of FcγR mediated phagocytosis and that SHP1 associates with CBL [24].

## Conclusions

In conclusion, we favor two potential mechanisms for the observed PKC-mediated, SHP-1 dependent downregulation of phagocytosis: **1**) PKC directly phosphorylates and activates SHP-1 resulting in augmented dephosphorylation of CBL and the inactivation of Rac-GTP activity **2**) PKC phosphorylates other substrates including CBL itself resulting in augmented interaction with 14-3-3 and altered substrate availability for SHP-1 or PTKs. Alternatively, PKC could directly alter the protein tyrosine kinase activity of an ITAM associated PTKs like SYK or a SFK resulting in decrease in CBL tyrosine phosphorylation or alter CBL availability as as substrate for PTKs. It should be noted, that this model would not be consistent with our results, the observed SHP-1 dependency of the desensitization under conditions of PMA stimulation or PKC inhibition (Figure 6). Finally, the results may further support a general molecular mechanism for ITAM receptor desensitization which predicts that serine threonine kinases (e.g. PKC, PKA, etc.) can augment the dephosphorylation and/or block the tyrosine phosphorylation of important cellular substrates and therefore disassemble adapter protein networks as negative regulators of small G protein and ITAM signaling networks. Future experiments are planned in these model systems to utilize CBL and SYK mutants and knockout mouse models to further validate these different hypotheses.

## Methods

### Antibodies and reagents

FcγR1 specific antibody [mAb 32.2 F(ab)_2_ frgment for IgG] was purchased from Medarex (Annandale, NJ). The cross linking Ab was a rabbit anti-mouse F(ab)_2_ fragment (R/M) obtained from Organon technika (West Chester, PA). Sheep red blood cells and rabbit IgG fraction to sheep RBC were purchased from ICN (Costa Mesa, CA). Anti-phosphotyrosine (4G10) was procured from Upstate Biotechnology (Lake Placid, NY). Anti-CBL and anti-CRKL were obtained from Santa Cruz biotechnology (Santa Cruz, LA). PKC activator, PMA (phorbol ester) and PKC inhibitor bisindolymaleiamide 1 (GF109203X) were from Calbiochem (San Diego, CA).

### Cell culture and activation

U937 cells were cultured in RPMI 1640 with 10% FBS and differentiated with 250U/ml human recombinant IFNγ for 5 days (termed U937IF) as described [73]. J774A1 cell line obtained from American type of culture collection was grown in DMEM supplemented with 10% FBS. For cross linking of FcγR1 of U937 cell, the cells (20 × 10^6^/500 μl in serum free RPMI) were incubated with anti FcγR1 [32.2 F(ab)_2_], 1 μg/0.5 ml at 37°C for 30 min. Cross-linking was done with rabbit F(ab)_2_ fragments to mouse IgG (5 μg/sample) at 37°C for different time periods. The reactions were terminated by centrifugation at 3000 rpm for 5 min. at 4°C after adding 800 μl pre-chilled HBSS. Cell pellet was lysed with 0.5 ml lysis buffer containing 1% Triton X-100 on ice for 30 min.. For stimulation and cross-linking of Fcγ receptor in J774A.1 cells, sheep RBC was sensitized with rabbit IgG fraction. 100 μl of pre-washed blood was added in 10 ml of PBS containing 10 μl of rabbit IgG fraction and incubated at room temperature for 1 hour with intermittent mixing followed by centrifugation at 1400 rpm for 5 min. at 4°C. After aspiration of supernatant, equal volume of serum free DMEM was added. For cross linking, cells were counted and adjusted to 3 × 10^6^ cells/ sample washed with HBSS and finally 1 ml of sensitized sheep RBC was added to each sample and incubated at 37°C for different time periods. Activation was stopped by centrifugation at 10,000 rpm for 1 min at 4°C. The cell pellet was lysed by adding 0.5 ml lysis buffer containing 1% Triton X100 and kept on ice for 30 min.

### Biochemical analysis

The immunoprecipitations were carried out from clarified clear cell lysate using lysis buffer containing 1% Triton X100, 10 mM Tris HCl, pH 7.6, 50 mM NaCl, 5 mM EDTA, 50 mM NaF, 0.1% BSA, 1% aprotonin, 2 mM sodium orthovanadate and 0.01 mM phenyl arsine oxide as described by Park et al. [11]. GST fusion protein pull-down experiments were performed as described by Kyono et al. [12] with little modification. In brief, 10 μg of fusion protein (CrKL-SH2) was added to 500 μl of sample and incubated for 90 min. at 4°C. The immune complexes were collected by pre-washed glutathione sepharose before resolving in 12.5% SDS-PAGE. Equivalent amounts of GST fusion proteins were used in each experimental group were confirmed by Coomassie blue staining of the gels after the transfer of protein.

The activation of small GTPase (total Rac and Rac2) following FcγR1 stimulation in U937 cells was measured using GST PAK1-CRIB domain pull down assay. In brief, this includes crosslinking of FcγR1 as described above followed by preparation of cell lysate which is incubated with GST-CRIB domain fusion protein (from Upstate Biotechnology, Lake Placid, NY) to bind only GTP bound Rac/Rac2. U937 cells were pre-treated with PMA or PKC inhibitor GF10902X. Immunoprecipates, pull down sample and whole cell lysate were resolved on 10–12.5% acrylamide-0.193% bis-acrylamide gels by SDS-PAGE [11,12].

### Vaccinia virus expression system

The recombinant vaccinia virus was generated, purified and titrated following the protocol as described by Kant et al. [24].

### Isolation of peritoneal macrophages

Thioglycolate derived peritoneal macrophages were obtained 4 days after intraperitoneal injection of 1.5 ml of thioglycollate medium (Sigma) in C57BL/6 mice. Peritoneal macrophages were isolated by flushing the peritoneal cavity 3 × 5 mL with RPMI, 10% FCS. An aliquot is counted using a hemocytometer. Macrophages are plated at an appropriate density (5 × 10^5^/well, Costar 6 well plate) and become adherent overnight. Adherent cells are washed the next day two times with PBS to remove nonadherent or only loosely adherent cells and used for phagocytosis assay.

### Phagocytic assay

Phagocytosis assay was performed on J774A.1 cells (2 × 10^5^/well, Costar 12 well plate) or peritoneal macrophages isolated from C57BL/6 mice (5 × 10^5^/well, Costar 6 well plate). The cells were treated with PMA (0.2 μg/ml and 0.4 μg/ml) alone or along with PKC inhibitor GF109203X (0.5 μM and 1 μM). In other set of experiments, cells were infected with empty vector recombinant vaccinia virus (pSc65) or recombinant vaccinia virus for the expression of wild type SHP-1 or catalytically dead SHP-1 at a multiplicity of infection (MOI) of 2 pfu/cell for 4 hours at 37°C in 5% CO_2_. After incubation, media was changed and cells were subjected to sheep RBC coated with rabbit anti-SRBC IgG. The target to effector ratio was kept at 100:1. DMSO treated cells served as control. After 30 min. of incubation at 37°C, cells were scrapped, cytospins were prepared, fixed and stained with Wright Giemsa stain (Dade, AG, Switzerland) and the slides were observed under microscope for rosette formation. The remaining cells were subjected to water shock to lyse the non-engulfed extracellular sheep RBCs. The cells were suspended in DMEM containing 20% FBS. Using cytospin, cells were spun down on glass slide, fixed and stained with Wright Giemsa stain. One hundred cells were counted for each slide and phagocytic index (PI) was calculated in triplicate. PI is calculated as % of phagocytic cells × average number of sheep RBC engulfed by each cell. In every experiment, recombinant viral load for control (cells infected with empty vector recombinant vaccinia virus) compared with experimental sample (cells infected with either wild type SHP-1 or catalytically dead SHP-1) was equivalent. The capacity of J774A.1 cells to form rosettes via Fcγ receptor was not altered by vaccinia virus. Rosette formation and phagocytosis did not occur in absence of sensitizing RBCs, which establishes the FcγR specificity of this response. As a control for effects of recombinant vaccinia virus, J774A.1 cells were tested for equal viral load by quantitation of β-galactosidase activity measured with X-gal as described by Kant et al. [24]. Briefly, 50 μl of 1% X-gal (Sigma, St. Louis, MO) was added to 400 μl cells (1 × 10^5^) suspension in DMEM (10% FBS) at 37°C. The blue colored supernatant was diluted 1:10 for the measurement of optical density at 595 nm in a spectrometer (Molecular Devices, California).

## Abbreviations

FcγRI: High affinity Fc receptor for IgG; PKC: Protein kinase C; PMA: Phorbol 12-myristate 13-acetate; SH2: src homology 2 domain; SH3: src homology 3 domain; sRBCs: sheep red blood cells.

## Competing interests

The authors declare that they have no competing interests.

## Author’s contributions

SJ, ARS and DLD designed the research; SJ, ARS and MZ conducted the research; SJ, ARS and DLD analyzed the research; SJ, ARS and DLD wrote the manuscript. DLD had primary responsibility for final content. All authors read and approved the final manuscript.
